# Dr. Donald Maclean

**Published:** 1903-09

**Authors:** 


Dr. Donald Maclean, of Detroit, died at his home from gastroenteritis, July 24, 1903. He was born in Seymour Township, Ont., in 1839, and was graduated from Edinburgh University in 1862. He practised medicine in Kingston, Ont., until 1870, excepting the years 1863-64, when he was a surgeon of volunteers in the United States Army. In 1870 he became professor of surgery at the University of Michigan, and held the chair until 1889. He was for a number of years chief surgeon of the Michigan Central and Grand Trunk railroads, and in 1894 was president of the American Medical Association. Dr. Maclean was an honorary Fellow of the American Association of Obstetricians and Gynecology, a surgeon of note and a man of affairs, gifted in speech, with many accomplishments. He was a teacher and a writer as well as an operator of distinction.

